# Precise Magnetic Stimulation of the Paraventricular Nucleus Improves Sociability in a Mouse Model of ASD

**DOI:** 10.1007/s12264-025-01444-x

**Published:** 2025-08-04

**Authors:** Sha Liu, Quyang Yang, Pengfei Zhu, Xuan Liu, Qingbo Lu, Jie Yang, Jingyao Gao, Hongbin Han, Zhijun Zhang, Ning Gu, Tao Tan, Jianfei Sun

**Affiliations:** 1 Jiangsu Key Laboratory of Biomaterials and Devices, School of Biological Science and Medical Engineering, Southeast University, Nanjing, 210009 China; 2 State Key Laboratory of Digital Medical Engineering, Southeast University, Nanjing, 210096 China; 3 Department of Biochemistry and Molecular Biology, Medical School of Southeast University, Nanjing, 210009 China; 4 Department of Neurology, Affiliated Zhongda Hospital, Research Institution of Neuropsychiatry, School of Medicine, Key Laboratory of Developmental Genes and Human Disease, Southeast University, Nanjing, 210009 China; 5 Nanjing Key Laboratory for Cardiovascular Information and Health Engineering Medicine, Institute of Clinical Medicine, Medical School, Nanjing Drum Tower Hospital, Nanjing University, Nanjing, 210093 China; 6 Shenzhen Key Laboratory of Precision Diagnosis and Treatment of Depression, Department of Mental Health and Public Health, Faculty of Life and Health Sciences, Shenzhen Institute of Advanced Technology, Chinese Academy of Sciences, Shenzhen, 518055 China; 7 Oujiang Laboratory (Zhejiang Lab for Regenerative Medicine, Vision and Brain Health), Institute of Aging, Key Laboratory of Alzheimer’s Disease of Zhejiang Province, Zhejiang Provincial Clinical Research Center for Mental Disorders, The Affiliated Wenzhou Kangning Hospital, Wenzhou Medical University, Wenzhou, 325000 China; 8 Department of Radiology, Beijing Key Laboratory of Magnetic Resonance Imaging Devices and Technology, Peking University Third Hospital, Beijing, 100191 China

**Keywords:** Superparamagnetic nanodrugs, Deep brain magnetic stimulation, Hypothalamus paraventricular nucleus, Oxytocin, Autism

## Abstract

Magnetic stimulation has made significant strides in the treatment of psychiatric disorders. Nonetheless, current magnetic stimulation techniques lack the precision to accurately modulate specific nuclei and cannot realize deep brain magnetic stimulation. To address this, we utilized superparamagnetic iron oxide nanoparticles as mediators to achieve precise targeting and penetration. We investigated the effects of magnetic fields with varying frequencies on neuronal activity and compared the activation effects on neurons using a 10-Hz precise magneto-stimulation system (pMSS) with repetitive transcranial magnetic stimulation in mice. Oxytocin levels, dendritic morphology and density, and mouse behavior were measured before and after pMSS intervention. Our findings suggest that pMSS can activate oxytocinergic neurons, leading to upregulation of oxytocin secretion and neurite outgrowth. As a result, sociability was rapidly improved after a one-week pMSS treatment regimen. These results demonstrate a promising magneto-stimulation method for regulating neuronal activity in deep brain nuclei and provide a promising therapeutic approach for autism spectrum disorder.

**Supplementary Information:**

The online version contains supplementary material available at 10.1007/s12264-025-01444-x.

## Introduction

Psychiatric and neurological disorders, such as autism spectrum disorder (ASD), are increasingly threatening global public health [1]. ASD is usually associated with complex circuits involving multiple cortical regions and nuclei; however, without an organic lesion, the underlying mechanism remains unclear [2, 3]. Owing to this characteristic, these diseases are difficult to treat with drugs or surgery. However, bioelectronic therapies directly act on critical nodes *via* an implanted electrical stimulator to play a therapeutic role in relevant diseases; this has attracted global interest from scientists [4–6]. Compared with electrical stimulation, magnetic stimulation has some advantages. For one thing, magnetic stimulation does not require surgical implantation [7]. For another, the human body is transparent to magnetic fields while the electrical fields readily diffuse *in vivo* [8]. In addition, magnetic stimulation can avoid unexpected electrochemical reactions occurring on the surface of an electrode [9]. Of more importance, the magnetic field is capable of inducing neuroplasticity by long-term stimulation rather than just improving symptoms [10]. Thus, magnetic stimulation is usually popular and takes priority in the clinic.

However, some aspects of magnetic stimulation remain in need of improvement, especially the penetration depth [11]. For example, repetitive transcranial magnetic stimulation (rTMS) that was approved by the U.S. Food and Drug Administration (FDA) for treatment of intractable major depressive disorder in 2008 [12], now plays a crucial role clinically [13–15]. The classic target for rTMS is the dorsolateral prefrontal cortex, which is now unable to satisfy the clinical demand. Recently, accumulating studies have revealed the underlying connections between the deep nuclei and the major psychiatric disorders [16–19], possibly bringing about innovative therapy with rTMS. However, the magnetic field can barely reach one deep nucleus, no matter how the coils are designed. This is due to the intrinsic inability of the magnetic field to achieve the focusing resolution and the penetration depth required. Therefore, this issue is impossible to solve just by the design of coils.

Regarding this issue, we proposed to apply the magneto-confinement effect of superparamagnetic iron oxide nanoparticles (SPIONs) to break through the restriction of the magnetic field. In this strategy, a mild (< 0.1 T) and non-focusing magnetic field was applied for stimulation, while the SPIONs were utilized to enhance the magneto-effect at the target. Here, the target of stimulation is dependent on the location of nanodrugs so that the drawbacks of magnetic fields can be overcome. We validated the effectiveness of this strategy in a mouse model of depression by precise stimulation of the left prelimbic cortex and the visual cortex [20, 21]. This strategy has already been demonstrated to enable the differentiation of two adjacent cortices in mice to facilitate the study of the underlying mechanism.

In this work, we for the first time realized the precise magneto-stimulation of the paraventricular nucleus (PVN) with a SPION-mediated strategy. The PVN, located in the hypothalamus, is an important nucleus in the neuroendocrine system. Oxytocin, a hormone secreted by the PVN, has attracted increasing attention from neuroscience, psychology, and psychiatry due to its crucial role in the regulation of behaviors [22, 23]. Recently, oxytocin was hypothesized to improve autism-like behavior based on clinical evidence, which possibly brings about an innovative therapeutic means for treating social deficits or even autism by magnetic stimulation of the PVN. Unfortunately, the current rTMS technique is unable to precisely act on this nucleus. We have demonstrated that SPION-mediated magneto-stimulation can precisely activate the PVN to regulate the secretion of oxytocin, which significantly improves the sociability of a mouse model of ASD.

## Materials and Methods

### Establishment of ASD Model Mice

All animal care and experimental procedures were approved by the Institutional Animal Care and Use Committee of Southeast University (Ethics code: 20210311002). Eight-week-old male and female C57BL/6J mice, weighing ~20–26 g, were obtained from Sino-British SIPPR/BK Lab Animal Ltd (Shanghai, China). Male mice were used for experiments involving neural activity activation, while female mice were used to breed VPA-induced ASD mouse models.

Pregnant female mice received a single intraperitoneal (i.p.) injection of 600 mg/kg sodium valproate (1069-66-5, Sigma-Aldrich) post-conception to induce autism-like behavior in the offspring. Each pregnant female produced six to nine pups; the day of birth was recorded as day 0. Pups were weaned and group-housed (3–5 mice/cage) by biological sex into cages on postnatal day 26 (PND26). Behavioral tests were conducted between 14:00 and 18:00, following a 120-min acclimation period in the testing room to minimize environmental stress. For detailed protocols and analyses, refer to Note S1 in the supplementary information. All animal care and experimental procedures strictly adhered to institutional ethical guidelines and were approved by the Institutional Animal Care and Use Committee of Southeast University (Ethics code: 20210311002).

### Cell Cultures

Neuro-2a cells (mouse neuroblastoma cells, N2a, zl-056516) were sourced from Wuhan Pricella Biotechnology Co. and cultured in Dulbecco’s modified Eagle’s medium (DMEM, 11965118, Gibco), supplemented with 10% fetal bovine serum (A5670701, Gibco), 100 U/mL penicillin, and 100 μg/mL streptomycin (15140122, Gibco). Upon reaching 80–90% confluency, SPIONs were added and co-incubated for 24 h.

For pyramidal hypothalamic neuronal culture, embryos were collected in a sterile environment, and brains were dissected to obtain hypothalamic tissue, which was placed in ice-cold dissection solution. The tissue was digested with 0.125% trypsin (25200072, Gibco) at 37°C for 10 min, then neutralized with three times the volume of DMEM containing 10% fetal bovine serum. The tissue was triturated into a single-cell suspension, centrifuged, and resuspended in Neurobasal medium with 2% B-27 supplement (17504044, Gibco). Cells were plated at a density of 1×10^5^ per mL and incubated under 5% CO_2_ at 37 °C, with medium changes every other day.

### Cytotoxicity Assessment of Superparamagnetic Nanoparticles

SPIONs were prepared at concentrations of 1, 2, 4, 8, and 16 mg/mL in sterile PBS. N2a cells were seeded in 96-well plates (density: 5×10^3^ cells/well) and allowed to adhere for 24 h. Then, SPION solutions (1 μL per well) were added to the culture medium and co-incubated with cells for 24 h at 37°C under 5% CO₂. After incubation, 10 μL CCK-8 reagent (Beyotime, China) was added to each well according to the manufacturer’s protocol. Plates were incubated at 37°C for 30 min, and absorbance was measured at 450 nm using a microplate reader. Cell viability (%) was calculated as:

$$ {\text{Viability}} = \left( {{\text{OD}}_{{{\text{Experimental}}}} {-}{\text{OD}}_{{{\text{Blank}}}} } \right)/({\text{OD}}_{{{\text{Control}}}} {-}{\text{OD}}_{{{\text{Blank}}}} ) \times {1}00\% . $$

### Construction of Precise Magneto-stimulation System (pMSS) Suitable for Mouse Brain

The pMSS system utilized γ-Fe_2_O_3_ SPIONs and a pair of disk-like magnets. The γ-Fe_2_O_3_ nanoparticles (brand name, Ferumoxytol) are approved by the FDA as an intravenous iron supplement. A rotating magnetic field was generated using an NdFeB (neodymium, iron, and boron) static magnet and a motor from Innuvo (China). For brain stimulation, mice were exposed to a 50 mT magnetic field, measured with a Gauss meter (HT20; Hengtong, China).

### Parameters for rTMS Modulation of N2a Neuronal Activity

The rTMS procedure was carried out using a CCY-I magnetic stimulator (Yiruide Medical Equipment Co., Ltd, Wuhan, China) equipped with a specially designed circular induction coil (model Y064). The coil exhibited concentric dimensions of 18 mm (inner diameter) and 57 mm (outer diameter), featuring a parallel-wound solenoidal configuration with multilayer winding architecture. Technical specifications included: axial height = 20.4 mm, conductor cross-sectional area = 18 mm^2^, and 30 total turns arranged in 6 vertically stacked layers (5 turns/layer) [24]. Stimulation parameters were configured as follows: The coil assembly was positioned 12 mm above the plane of the N2a cell culture substrate. High-frequency repetitive TMS was delivered at 10 Hz with intensity calibrated to 50% of the device's maximum output (2.60 T measured at a coil surface contact point). Each stimulation protocol comprised 50 burst trains, with individual sequences containing 10 pulses followed by 9 s inter-train intervals, yielding 500 total pulses per experimental session.

### Calcium Imaging

Ca^2+^ ion endocytosis under pMSS was monitored using Fluo-4 AM (S1060, Shanghai Biyuntian Biotechnology Co.), a fluorescent probe for Ca^2+^ ions. Fluo-4 AM, which emits green fluorescence upon hydrolysis by intracellular esterases, was used to stain N2a cells. N2a cells were incubated with Fluo-4 staining solution at 37°C for 30 min in the dark, and Ca^2+^ flow was dynamically recorded under a fluorescence microscope (Eclipse Ti2, Nikon, Fluo-4 AM excitation/emission = 490/525 nm).

Ca^2+^ images were processed using MATLAB (MathWorks). The Ca^2+^ trace of each active neuron was extracted by defining a circular region of interest (ROI) with a 15 mm diameter, centered on the detected cell position. The average fluorescence of the surrounding circular region was used as the baseline background fluorescence (F<ring>). The Ca^2+^ signal of each mouse (Fsig) was calculated by subtracting the baseline background fluorescence (F<ring>) from the fluorescence of the ROI (F<ROI>): Fsig = F<ROI> − F<ring>. Each Ca^2+^ trace ΔF/F_0_ was calculated as Fsig divided by the baseline fluorescence over the neuron ROI (F_0_). F_0_ was estimated by subtracting the original Ca^2+^ signal F<ROI> from the video background luminance curve (Fb), and Fb was formed by considering the minimum value of the fluorescence of each frame of the video. Given that the signal may contain multiple Ca^2+^ transients, F_0_ was determined using the 15% quantile instead of the mean, i.e., F_0_ = F<ROI> − Fb.

### Virus Injection and Fiber/GRIN Lens Implantation

Under anesthesia, mice were positioned on a stereotaxic apparatus (71000-S, RWD Life Science). The virus was injected using a modified syringe at a rate of 0.1 μL/min, with the needle remaining in place for 5 min post-injection. A total of 0.5 μL of rAAV-hSyn-GCaMP6f (BC-0079, Braincase) or AAV9-hSyn-OT1.0 (PT-2741, BrainVTA) was bilaterally injected into the target area, with coordinates (mm from bregma): PVN: −0.82 anteroposterior (AP); ±0.25 mediolateral (ML); −4.85 dorsoventral (DV). Two weeks following the surgery, SPIONs were delivered into the PVN at the same coordinates. An optical fiber (400-µm, FOC-W-1.25-400, Inper) or GRIN lens (1-mm, GT-LFRL-100-025-50-NC, GRINTECH GmbH) was inserted into the injection site and secured using adhesive dental cement (S380, C&B Metabond).

### Fiber Photometry and MiniScope Recordings

Three weeks after GRIN lens implantation, the miniscope was attached to the mouse's head and adjusted for clear focus across the field of view. The miniscope body was detached and reattached to the base for each imaging experiment using a side-locking screw. During experiments, Ca^2+^ activity at the single neuron level in the PVN was recorded as the mice underwent pMSS.

### *In Vivo* Microdialysis

Mice were anesthetized with pentobarbital and isoflurane, then surgically implanted with a guide cannula (AG-X, Eicom, Japan) into the PVN. After surgery, mice were allowed to recover for five days. On the sixth day, microdialysis was applied using a probe (FZ-X-Y, Eicom, Japan) at 1.5 μL/min. Artificial cerebrospinal fluid of the following composition was used (in mmol/L): 147 NaCl, 2.7 KCl, 1.2 CaCl_2_, 0.85 MgCl_2_, pH 7.4. Samples were collected and stored at −80 °C until testing for oxytocin secretion using an ELISA kit (E-EL-0029, Elabscience Biotechnology, China).

### Oxytocin Administration

Oxytocin (HY-17571A, Tocris Bioscience) was administered either intravenously (20 mg/kg) [25] or by local infusion into the PVN. The mice in this experiment weighed ~25.5 g, so ~510 μg of oxytocin was injected into each mouse *via* the tail vein. Since the mass of the PVN of C57BL/6J mice couldn't be measured and no relevant literature was available, the mass of the hypothalamus was taken as the reference for *in situ* oxytocin injection. Given that the PVN mass of adult C57BL/6J mice is ~10 mg, ~0.2 μg of oxytocin was injected into the PVN of each mouse.

### Administration of Oxytocin Receptor Antagonists

Oxytocin receptor antagonists (MCE, L-372662) were delivered to the medial prefrontal cortex (mPFC; coordinates (in mm): AP +2.0, ML ±0.3, DV −2.0) and lateral ventricle (LV; coordinates: AP −0.6, ML ±1.0, DV −2.0) of ASD mice *via* stereotaxic surgery. The injection protocol followed previously established parameters [26], with a concentration of 2 μg/μL and a total volume of 1 μL per site.

### Histology

Following the injection of magnetic nanoparticles into the PVN (AP −0.82, ML ±0.25, and DV −4.5) using a modified needle, mice were allowed to recover for 1 week before sacrifice. The mice were anesthetized (i.p.) and perfused with 50 mL of 4% paraformaldehyde (PFA) in phosphate-buffered saline (PBS) *via* intracardiac puncture. Brains were then post-fixed in 4% PFA at 4°C for 24 h, then dehydrated in 30% sucrose. Sections were cut at 5 μm and stained with hematoxylin and eosin (H&E; C0105S, Beyotime) and a Prussian Blue Iron Stain Kit (G1420, Solarbio).

For immunofluorescence, 50 μm-thick sections were mounted on slides and permeabilized with 0.5% Triton X-100 in Tris-buffered saline (TBS). Then they were incubated with primary and secondary antibodies as required, and the nuclei were stained with 6-diamidino-2-phenylindole (DAPI). Images were captured using an FV1200 confocal microscope (Olympus). Antibody details are listed in Table S1 in the Supplementary Information. In addition, the percentage of c-Fos positive cells was calculated using the following formula: percentage of c-Fos positive cells = (number of positive cells/ total DAPI-stained nuclei) ×100%.

For Golgi-Cox staining, the FD Rapid GolgiStain™ Kit (PK401, FD NeuroTechnologies) was used. Brain tissues were soaked in an AB mixture (A + B, 1:1 ratio, 10 mL/mouse) for 21 days, followed by soaking in C solution for 72 h. Slices were cut at 150 μm at −80°C and mounted on gelatin-coated slides. Tissue dehydration was achieved through a gradient of ethanol concentrations (50%, 75%, 95%, and 100%). Transparency was attained using xylene, and slides were sealed with neutral resin. Dendritic spine images were captured with a 63× objective lens (Zeiss Axio Observer 7, Germany). Dendritic spine density in PVN neurons was quantified using ImageJ software; ~8 neurons were analyzed from 3 mice in a blinded manner.

### RNA-Sequencing

After 7 days of continuous pMSS, the PVN of the mouse brain was collected for RNA sample preparation. Three mice from each experimental group (pMSS-OFF/pMSS-ON) were used. RNA sequencing was carried out by Lianchuan Biotech (Hangzhou, China). In brief, RNA was reverse-transcribed into cDNA, fragmented, and specific adapters were attached to form a sequencing library. This library was sequenced using a high-throughput sequencer, generating extensive sequence data. Quality-filtered data underwent bioinformatics analysis, including functional annotation, gene enrichment, and transcription factor analysis. Differentially-expressed genes (*P* < 0.05, Student's *t* test) were identified and classified by expression profiles. Functional enrichment analysis was applied using Gene Ontology (GO) (http://www.geneontology.org/) and Kyoto Encyclopedia of Genes and Genomes (KEGG) pathways (https://www.kegg.jp/kegg/pathway.html).

### Quantitative Real-Time Polymerase Chain Reaction (qRT-PCR)

Total RNA was extracted from mouse brain using the TRIzol reagent (15596026CN, Invitrogen) in accordance with the manufacturer's instructions. Subsequently, cDNA synthesis was carried out using the HiScript^®^ III RT SuperMix (R222-01, Vazyme) for qPCR (+gDNA wiper) reverse transcription kit (Vazyme) with 1 mg of total RNA. A qRT-PCR was performed using the ChamQTM SYBR^®^ qPCR Master Mix kit (Q311-02, Vazyme) on a Step OnePlus thermal cycler (4376600, Applied Biosystems). The expression levels of the target genes were normalized to glyceraldehyde 3-phosphate dehydrogenase as the reference gene. The primer sequences for the target genes can be found in Table S1. Each experiment was repeated six times to ensure the reliability and reproducibility.

### Western Blot

For protein analysis, a 5% concentrated gel and a 10% separation gel were prepared, and each gel lane was loaded with 40 μg of total protein. After electrophoretic separation, proteins were transferred onto a polyvinylidene fluoride membrane. The membrane was rinsed with Tris Buffered Saline with Tween (TBST) for 5 min and blocked with TBST containing 5% bovine serum albumin (BSA) for 1 h. The corresponding primary antibody diluted 1:1000 in TBST solution with 5% BSA was added and incubated overnight at 4°C. The membrane was then rinsed three times for 10 min each with TBST. Thereafter, the corresponding labeled secondary antibody diluted 1:1000 in PBS was added and incubated for 2 h at room temperature. The membrane was subsequently rinsed three times with TBST for 10 min each. Finally, densitometric analysis was applied using TotalLab software, and the experiment was repeated three times to ensure the reliability and reproducibility of the results. Information regarding the antibodies used can be found in Table S1 in the Supplementary Information.

### Quantification and Statistical Analysis

Data were analyzed using one-way or two-way analysis of variance (ANOVA), followed by *post hoc* tests for multiple comparisons or *t* tests. Results are presented as the mean ± standard error of the mean. A statistically significant difference was set at *P* < 0.05, with asterisks denoting significance levels (*P < 0.05, **P < 0.01, *** P < 0.001, ^###^* P* < 0.001).

## Results

### Materials and Equipment of the Precise Magnetic Stimulation System (pMSS) for Neuronal Activation* In Vitro*

**Fig. 1:**
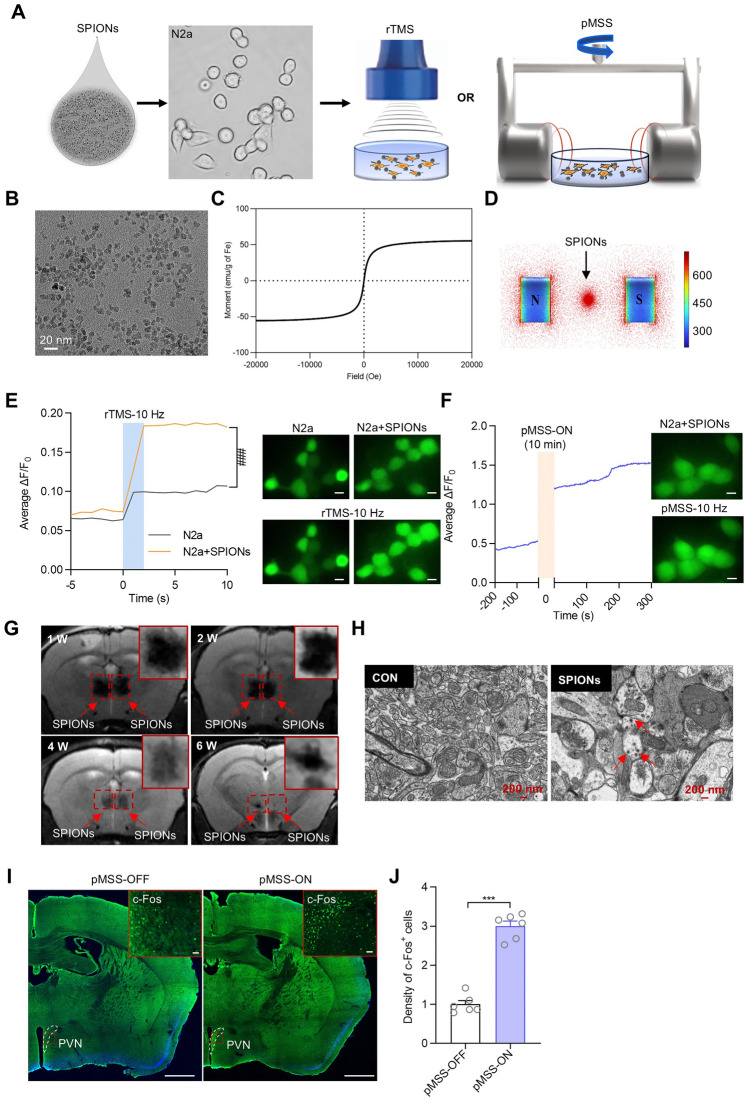
pMSS efficiently activates neuronal activity. **A** Diagram showing neuronal stimulation with rTMS or pMSS of N2a cells with SPIONs. **B** TEM images showing SPIONs (scale bar, 20 nm). **C** Hysteresis loop of the SPION solution. **D** Simulation of the magnetic flux distribution experienced by SPIONs in a static magnetic field. **E** ΔF/F_0_ of N2a cells responding to rTMS with or without SPIONs [two-way ANOVA: *F* (1, 15) = 31.60]. Insets: representative images of N2a cells loaded with Fluo-4 AM before (upper) and after (lower) 10 Hz rTMS (scale bar, 20 μm; *n =* 15 cells). **F** ΔF/F_0_ of N2a cells containing SPIONs responding to 10 Hz-pMSS. Insets: representative images of N2a cells before (upper) and after (lower) pMSS (scale bar, 20 μm; *n* = 14 cells). **G** MRI images of SPIONs over time in the PVN of mice [W, week(s)]. **H** TEM images showing SPIONs in PVN slices treated with SPIONs for 14 days (scale bar, 200 nm). **I** Representative images of PVN c-Fos immunofluorescence (coronal section) after 10 min of pMSS (scale bars, 50 μm, 1000 μm). **J** c-Fos immunopositive cell counts in the PVN (*n =* 6 mice). All data are represented as the mean ± SD, ^###^*P* < 0.001, ^***^P < 0.001, two-tailed Student’s *t* tests.

The pMSS in our experiments comprised superparamagnetic γ-Fe_2_O_3_ nanoparticles (SPIONs) and a rotational magnetic field of mild intensity (Fig. 1A). The nanoparticles were capped by polyglucose sorbitol carboxymethylether, and the size of the nanoparticles was ~20 nm (Fig. 1B). The magnetic hysteresis loop indicated that the SPION solution was superparamagnetic. The saturation magnetization was 55.43 electromagnetic units/g (Fig. 1C). Detailed information about this nanomaterial is available in Supplementary Note S2. The magnetic field was produced by a pair of disk-like magnets with a spacing of 4 cm and a cross-sectional area of 25 cm^2^. The intensity of the magnetic field in the central region was ~50 milliTesla (mT). The field distribution was simulated using COMSOL software (COMSOL, Sweden) based on the actual parameters, revealing near-uniformity within the central region (Fig. 1D).

With this system, the optimal concentration of γ-Fe_2_O_3_ nanoparticles was first determined using a cell viability assay with N2a cells following a 24-h incubation. We found that 2 mg/mL was the optimum concentration (Fig. S1A). This fixed concentration of nanoparticles was maintained in subsequent experiments. Next, the effect of rTMS combined with SPIONs on Ca^2+^ influx in N2a cells was compared with standalone rTMS. It was shown that the rTMS combined with SPIONs increased the mean ΔF/F_0_ values of neurons to twice the baseline values compared with the standalone rTMS (Fig. 1E), meaning the presence of magnetic nanomaterials can enhance the biological effect of magnetic stimulation. Then, the Ca^2+^ influx in N2a cells was also investigated using the pMSS at 1 Hz, 5 Hz, and 10 Hz. The results revealed that the 1 Hz SPION-mediated magnetic stimulation slightly inhibited the Ca^2+^ transients of N2a cells (Fig. S1B), while 5 Hz magnetic stimulation had little significant effect on neuronal Ca^2+^ activity (Fig. S1C). Interestingly, the 10 Hz magnetic stimulation significantly enhanced the Ca^2+^ influx in N2a cells (Fig. 1F). Thus, by adjusting the frequency, the SPION-mediated magnetic stimulation can modulate neuronal activity in a bidirectional manner, causing either excitatory or inhibitory effects on neurons.

### Neural Activation by SPION-Mediated Magnetic Stimulation in Wild-Type Mice

Firstly, the residence stability of SPIONs in the brain was determined by magnetic resonance imaging (MRI), which verified the presence of SPIONs for at least four weeks without significant diffusion (Fig. 1G). In addition, transmission electron microscopy (TEM) analysis of brain sections after 14 days revealed cellular internalization of SPIONs (Fig. 1H). Furthermore, injection of SPIONs mixed with cholera toxin subunit B (CTB) into the right PVN, followed by Perl’s staining and H&E staining (Fig. S1D, E), confirmed the accuracy of delivery and safety of SPIONs. Importantly, it was found that the SPION-mediated magnetic stimulation successfully upregulated c-Fos expression in the PVN while the superficial cortical activity was scarcely affected (Fig. 1I, J). Furthermore, a single 10-min 10-Hz pMSS session elicited robust neuronal activation in the PVN, with the elevated excitatory state persisting for at least 5 h post-stimulation, as quantified by longitudinal c-Fos⁺ neuronal density analysis (Fig. S2).

**Fig. 2:**
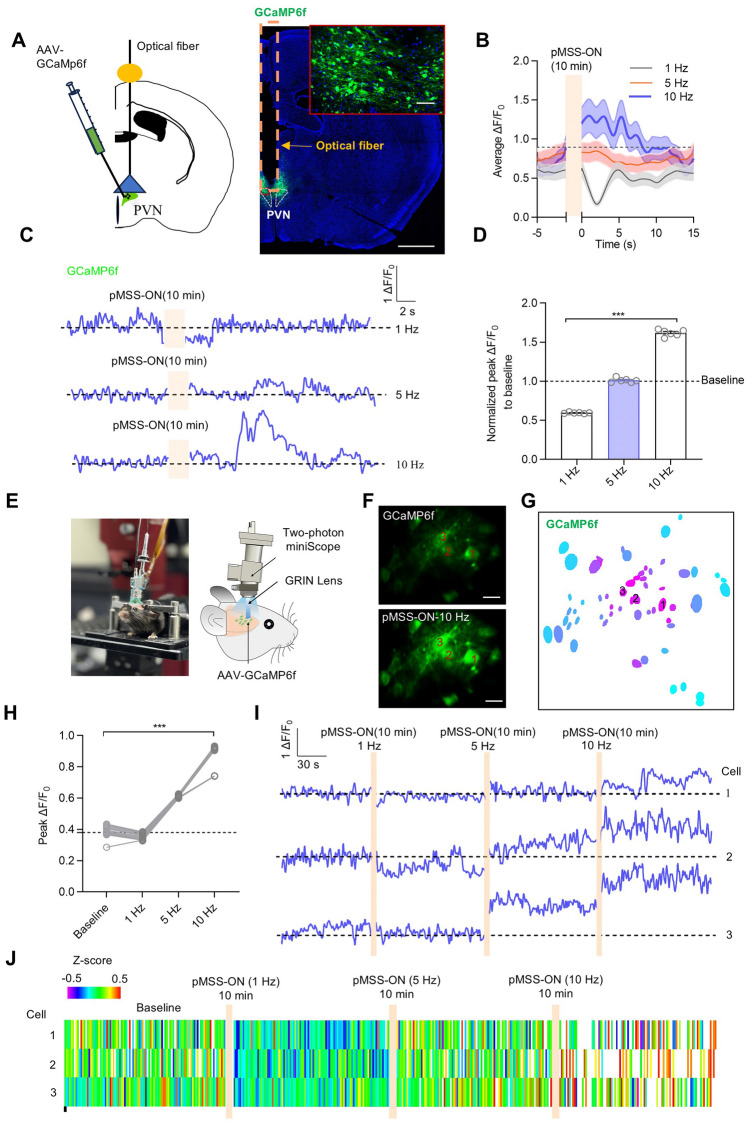
pMSS activates neuronal activity in the PVN among the deep brain nuclei. **A** Representative image of a brain slice (coronal section) injected with GCaMP6f (scale bars, 50 μm, 1000 μm). **B** Plots of average ΔF/F_0_ of neurons in the PVN responding to pMSS of different frequencies (*n =* 6 mice). **C** ΔF/F_0_ of neurons in the PVN responding to pMSS of different frequencies. **D** Normalized peak ΔF/F_0_ in the PVN responding to pMSS (*n =* 6 mice per group, one-way ANOVA: *F* (2, 15) = 1828). **E** Actual experimental setup and schematic illustration of the experimental design for two-photon Ca^2+^ imaging. **F** Representative images of two-photon Ca^2+^ in PVN (coronal section; scale bar, 20 μm). **G** Cell fields under pMSS in the PVN. **H** Peak ΔF/F_0_ of neurons in the PVN responding to pMSS (*n =* 3 cells, repeated measures one-way ANOVA: *F* (1.290, 18.92) = 869.2). **I** Representative raw Ca^2+^ traces of neurons marked in **H**. **J** Heatmaps of normalized Z-score in PVN neurons responding to pMSS of different frequencies (*n =* 3 cells). All data are represented as the mean ± SD, ^***^P < 0.001.

In addition, the neural activation of the right PVN was further investigated by monitoring Ca^2+^ transients *via* real-time fiber photometry. Signals were obtained from mice after a 10-min continuous magnetic stimulation using the genetically-encoded Ca^2+^ indicator GCaMP6f. The ΔF/F_0_ of neurons was calculated to assess the activation, which aligned with the findings in N2a cells (Fig. 2A). Magnetic stimulation at 1 Hz reduced the neuronal Ca^2+^ influx, while 5 Hz stimulation caused a slight increase in neuronal Ca^2+^ influx. As anticipated, the 10 Hz magnetic stimulation effectively activated PVN neuronal activity, as indicated by the change in ΔF/F_0_ (Fig. 2B–D).

To investigate the neuromodulatory effects of pMSS, we applied two-photon microscopy to record Ca^2+^ dynamics in PVN excitatory neurons following a 10-min magnetic stimulation (Fig. 2E–G). As demonstrated in Video S1-3, Ca^2+^ transients in individual PVN neurons exhibited frequency-dependent responsiveness. Specifically, low-frequency (1 Hz) magnetic stimulation induced significant neuronal inhibition, whereas high-frequency pMSS elicited activation of PVN neurons. Among tested frequencies, 10 Hz-pMSS demonstrated the most pronounced activation effect, with its peak ΔF/F_0_ value reaching nearly twice that of the baseline (Fig. 2H). Consistent with the Ca^2+^ transients, 1 Hz SPION-mediated magnetic stimulation slightly suppressed the activity of mouse-PVN neurons, as demonstrated in the average ΔF/F_0_ trace plot and the corresponding heatmap. In contrast, 5 Hz stimulation showed a tiny activation effect for the mouse PVN neurons (Fig. 2I, J). Overall, our findings validate the long-lasting stability and cellular uptake of SPIONs within the brain, affirming their safe and precise delivery into the PVN *in vivo*. Furthermore, our findings revealed the frequency-dependent feature of neuronal activation, providing further evidence for the bidirectional modulation of neuronal activity by pMSS.

### Precise Magnetic Stimulation of the PVN Promotes Oxytocin Release by Activating Oxytocinergic Neurons

**Fig. 3:**
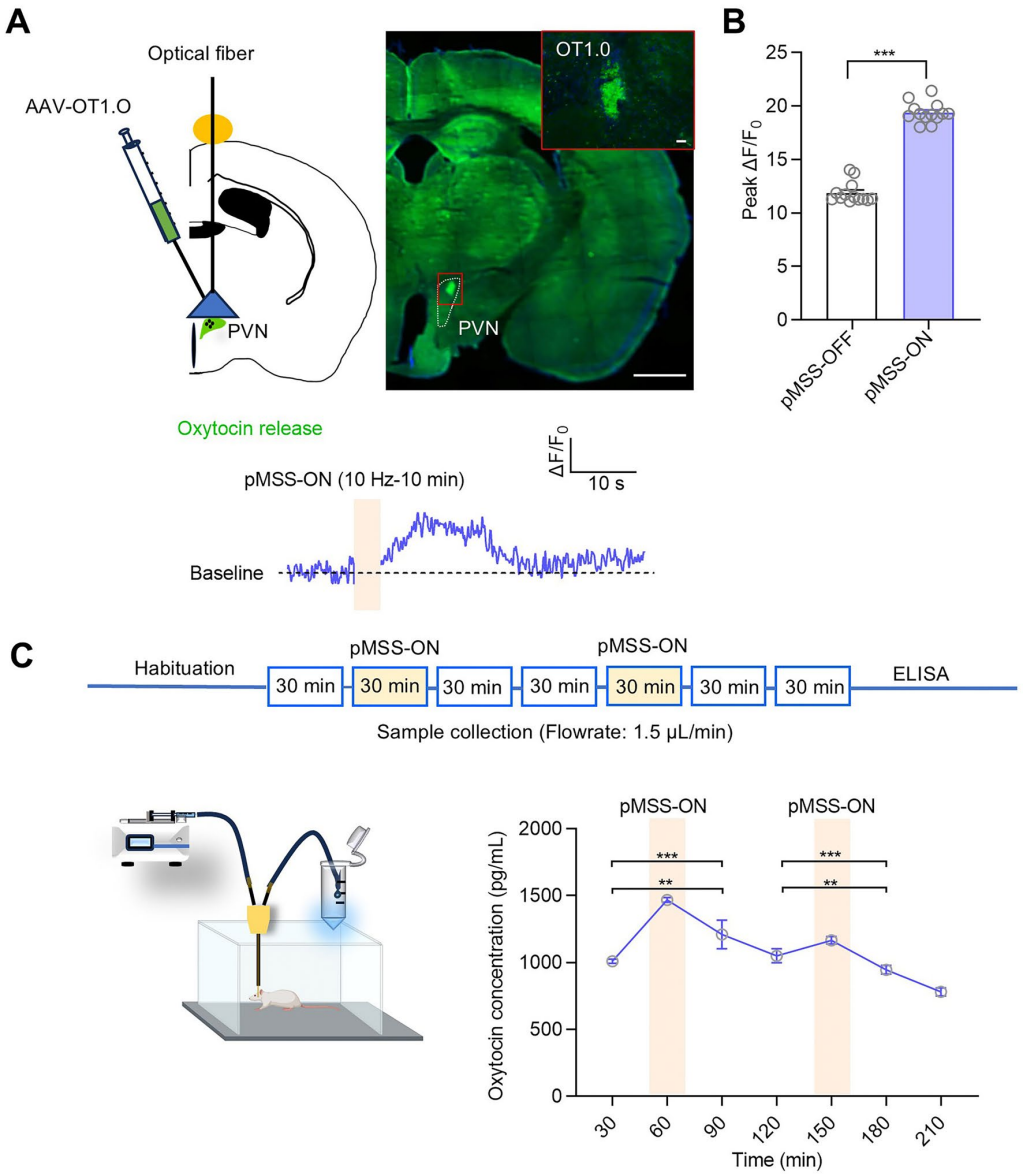
pMSS activates oxytocinergic neurons to promote oxytocin release. **A** Representative fluorescence image of oxytocinergic neurons loaded with OT1.0. Average ΔF/F_0_ trace of OT1.0 in the PVN responding to 10-Hz pMSS (*n =* 5 mice). **B** Peak ΔF/F_0_ in the PVN responding to 10-Hz pMSS (*n =* 13, paired two-tailed Student’s *t* test). **C** Scheme of microdialysis *in vivo*; level of oxytocin release from the PVN of mouse brain under 10 Hz-pMSS (*n =* 6 mice per group, one-way ANOVA: *F* (2, 15) = 79.42, *F* (2, 15) = 47.79; two-tailed Student’s *t* test, *P* = 0.001 between 30 min and 90 min, *P* = 0.002 between 120 min and 180 min). All data are represented as the mean ± SD, ^**^P < 0.01, ^***^P < 0.001.

Given the role of the PVN in oxytocin secretion, the activation of oxytocinergic neurons was further investigated. Adeno-associated viruses (AAVs) encoding OT1.0 (hSyn-OT1.0) were injected into the PVN of wild-type mice, and a 10-Hz SPION-mediated magnetic stimulation was applied after three weeks. We observed that the magnetic stimulation led to a significant increase of OT1.0 fluorescence in the PVN (Fig. 3A). The mean ΔF/F_0_ footprint plots of OT1.0 indicated an increase in oxytocin release by the 10-Hz stimulation, with the peak release reaching nearly twice the baseline value (Fig. 3B). The dose-response curve of stimulation time demonstrated oxytocin release enhancement of up to 1500 pg/mL within 30 min of continuous magnetic stimulation (Fig. 3C).

**Fig. 4:**
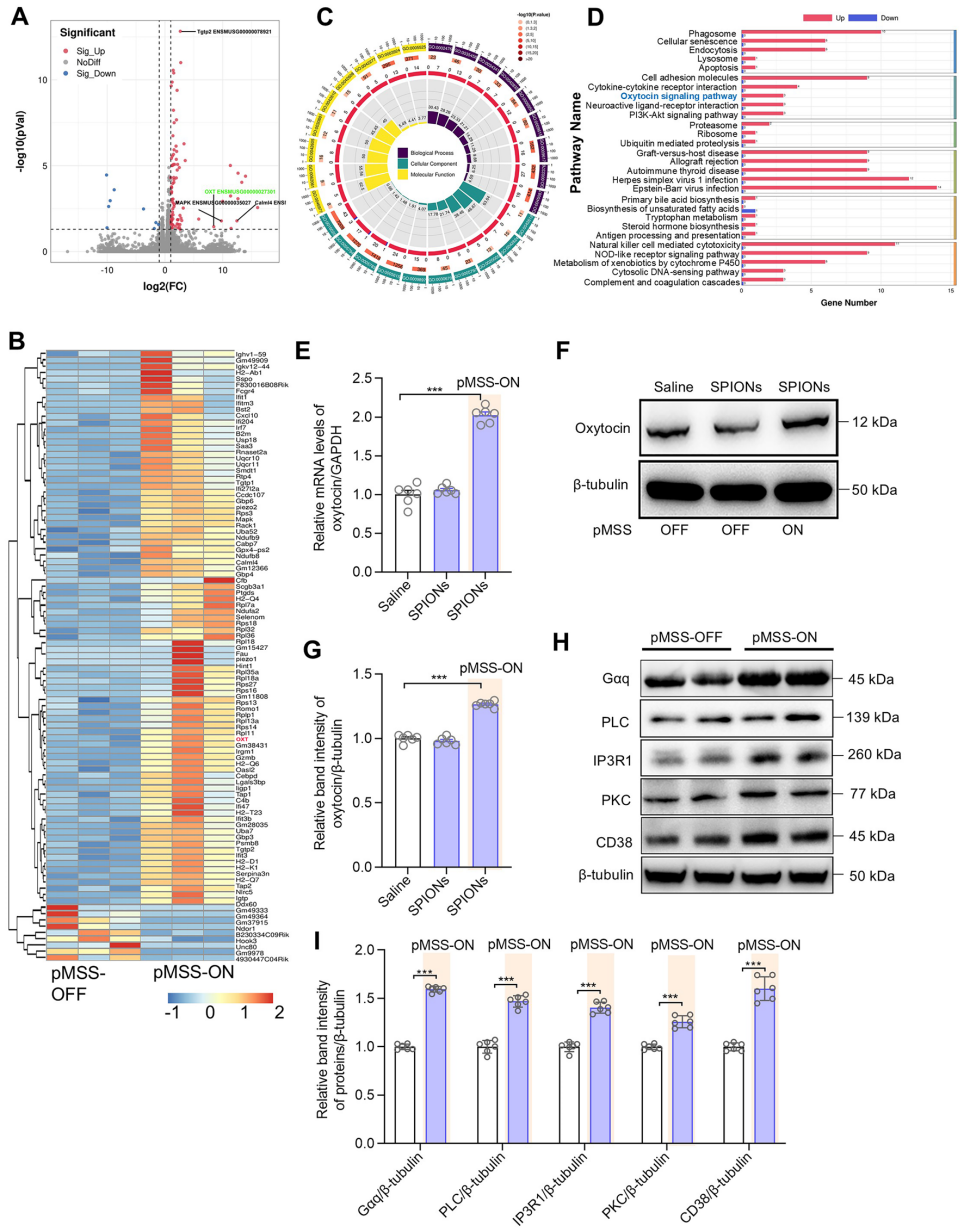
pMSS causes significant differences at the transcriptome level in the mouse PVN. **A** Volcano map of differentially-expressed genes. **B** Heat map of differential gene clustering. **C** GO enrichment circle plot for differentially-expressed genes. **D** KEGG enrichment bar-plot; horizontal coordinate, the number of differential genes included in the pathway; vertical coordinate, the pathway name. **E** The qRT-PCR results for oxytocin in the PVN after 7 days of pMSS (*n =* 6 mice per group, one-way ANOVA: *F* (2, 15) =161.9). **F** Representative immunoblots of oxytocin in the PVN. **G** Relative expression levels of oxytocin in the PVN after 7 days of pMSS (*n =* 6 mice per group, one-way ANOVA: *F* (2, 15) =54.79). **H** Representative immunoblots of the main proteins in the oxytocin signaling pathway within the PVN. **I** Relative fold-change in protein expression in the PVN after 7 days of pMSS (*n =* 6 mice per group, two-tailed Student’s *t* test). All data are represented as the mean ± SD, ^***^P < 0.001.

Furthermore, we utilized participatory transcriptome sequencing to identify differentially-expressed genes in the transcriptome of SPION-mediated magnetic stimulation of the PVN. The volcano plot of the differentially expressed genes revealed 88 up-regulated genes and 9 down-regulated genes (Fig. 4A). Fig. 4B shows a heat map of the relative expression levels of specific differentially-expressed genes in the experimental groups. Moreover, the SPION-mediated magnetic stimulation of the PVN led to differential expression of the transcriptome in the mouse brain. The GO enrichment circle plot and KEGG enrichment bar plot confirmed this result. This differential expression significantly affected the function of the oxytocin signaling pathway (main signaling axis: Gαq-PLC-IP3R1-PKC-CD38) (Fig. 4C, D).

To further validate the results of the signaling pathway analysis, we conducted RT-qPCR and Western blot assays to examine the expression levels of key genes and proteins involved in the oxytocin signaling pathway. Our findings revealed a nearly 2-fold increase in oxytocin mRNA expression under 10-Hz stimulation compared to the saline group (Fig. 4E). In addition, western blot results also demonstrated that the oxytocin expression was enhanced by the same treatment conditions (Fig. 4F, G). Furthermore, we investigated the relative expression levels of key proteins in the oxytocin signaling pathway, showing that 10-Hz stimulation significantly increased Gαq, PLC, IP3R1, PKC, and CD38 expression levels (Fig. 4H, I), thereby confirming the activation of the oxytocin signaling pathway. These findings demonstrated the precise delivery of nanoparticles into the PVN, as well as the efficacy of magnetic stimulation at 10 Hz in activating neurons within the same nucleus. This is evidenced through the oxytocin release and alterations in gene expression patterns in the mouse brain.

### Precise Magnetic Stimulation of the PVN Improves Neurite Outgrowth

**Fig. 5:**
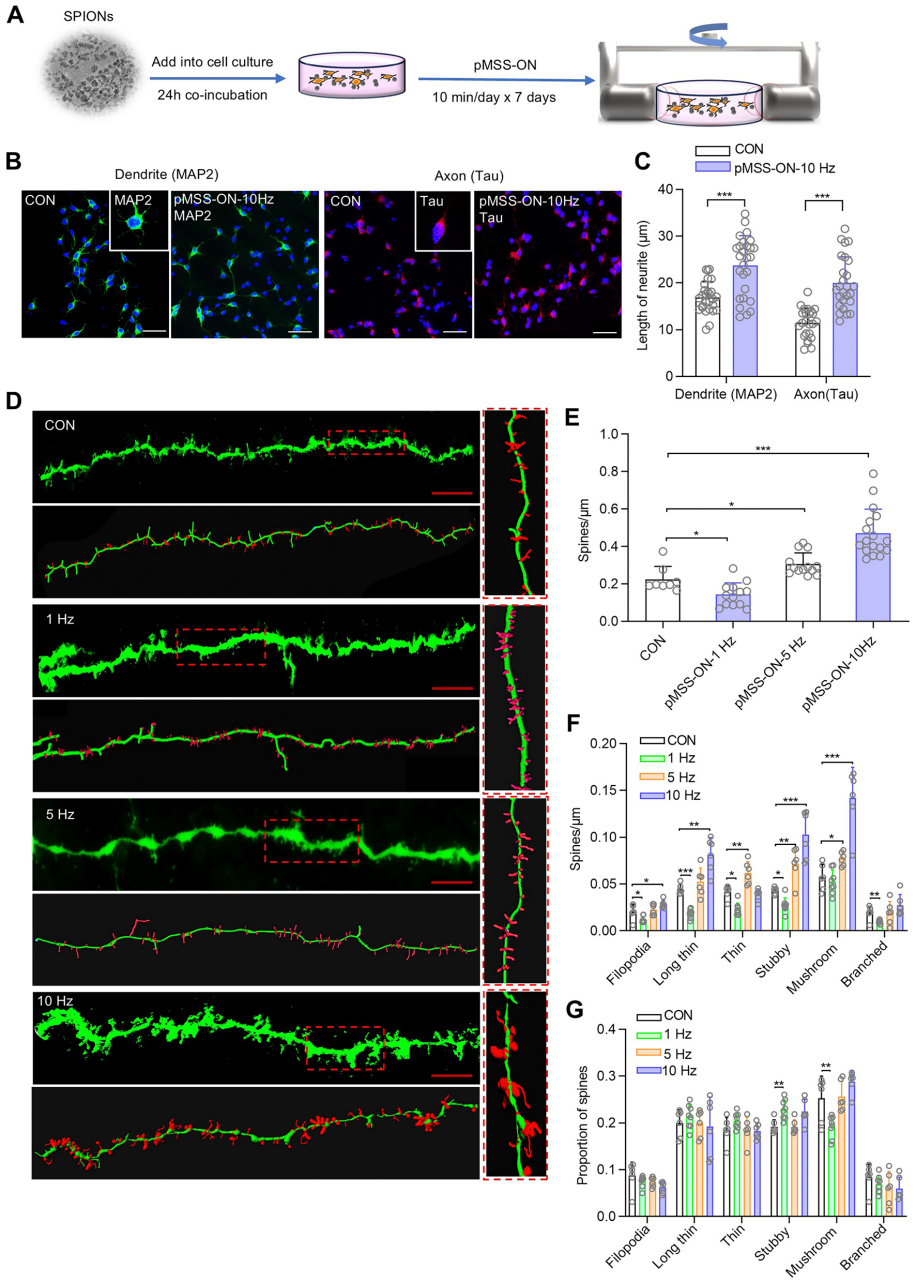
Neurite outgrowth is enhanced by 10-Hz pMSS in the PVN of wild-type mice. **A** Schedule of *in vitro* experiment design. **B** Immunofluorescence image of a primary neuron in the PVN (scale bar, 50 μm). **C** The dendrite and axon lengths measured in stained cells. Quantitative data are presented as length (μm), with two-tailed Student’s *t* tests. **D** Representative image of spines in the dendrites of neurons within the PVN. *n =* 6–10 neurons from three mice per group. Scale bar: 20 μm. **E** Plot of spine density, two-tailed Student’s *t* tests. **F** Plot of different types of spine density, two-tailed Student’s *t* tests. **G** Bar graph of proportion of different types of spines, two-tailed Student’s *t* tests. All data are represented as the mean ± SD, ^*^P < 0.05, ^**^P < 0.01, ^***^P < 0.001.

Previous research using high-throughput sequencing has indicated that precise 10-Hz magnetic stimulation effectively activates the PI3K-Akt signaling pathway that is known for promoting neurite growth and differentiation [27, 28]. Hence, we investigated the impact of pMSS on the growth and development of neurons. The primary PVN neurons received continued 10-Hz pMSS for 7 days *in vitro*. It was demonstrated by immunofluorescence staining that this treatment significantly enhanced both the neuron dendrite and axon growth (Fig. 5A–C).

Then we applied dendritic spine density analysis to PVN neurons after SPION-mediated magnetic stimulation to investigate the effect on neuronal development and function. Morphometric analysis of Golgi-Cox-stained neurons (Fig. 5D) demonstrated that 1-Hz stimulation on the PVN led to a reduction in total dendritic spine density compared with the control. However, high-frequency stimulation (5 Hz and 10 Hz) was able to reverse this trend, resulting in up to a 2-fold increase in dendritic spine density in the case of the 10-Hz treatment (Fig. 5E). In addition, our findings confirmed that the SPION-mediated magnetic stimulation increased the density of filopodia, long thin, thin, stubby, and mushroom dendritic spines but had little influence on the branched dendritic spines under 10-Hz stimulation (Fig. 5F). Specifically, 1-Hz stimulation induced an increase in the percentage of stubby dendritic spines and a significant decrease in the percentage of mushroom dendritic spines, whereas neither the 5-Hz nor the 10-Hz stimulation exhibited this phenomenon (Fig. 5G). These results revealed the potential of SPION-mediated magnetic stimulation in regulating synaptic plasticity.

### Secretion of Endogenous Oxytocin Rapidly Improves Sociability in Mice

Considering the role of oxytocin in neural regulation, the case that SPION-mediated magnetic stimulation can activate oxytocinergic neurons provides evidence to treat some psychiatric disorders by magnetic therapy. We established an ASD mouse model using valproic acid (VPA) modeling [29, 30], as outlined in Fig. S3. The VPA-induced mouse pups exhibited low body mass, delayed eye-opening, latency of righting reflexes, and short hanging time at age 5–6 weeks. At age 10–11 weeks, the ASD mouse model exhibited low active sociability, increased anxiety, and repetitive stereotyped behaviors, as evidenced by the three-chamber sociability, open-field, and self-grooming tests, respectively. This comprehensive assessment confirmed the suitability of the ASD mouse model for further experimentation.

Subsequently, we investigated the dosage-response relationship between the SPION-mediated magnetic stimulation and the oxytocin secretion by measuring the oxytocin levels in peripheral blood with different amounts of SPIONs and field intensities. Building on the results of prior experiments, 10-Hz stimulation can effectively activate neuronal activity, boost the release of oxytocin, and enhance dendritic spine density; thus, the 10-Hz frequency was applied in subsequent experiments. Our findings revealed that the SPION-mediated magnetic stimulation was based on the synergic effect of the magnetic field and SPIONs, as demonstrated by comparison with Au nanoparticles (Fig. S4A). Specifically, the oxytocin levels were positively correlated with both the SPION dosage and the magnetic field intensity (Fig. S4B, C). Under the optimized parameters with a magnetic field intensity of 50 mT, the oxytocin in peripheral blood could reach 17 ng/L at a mass of 0.4 μg of SPIONs in the PVN, and this causes a 1.5-fold increase compared to the saline group.

**Fig. 6:**
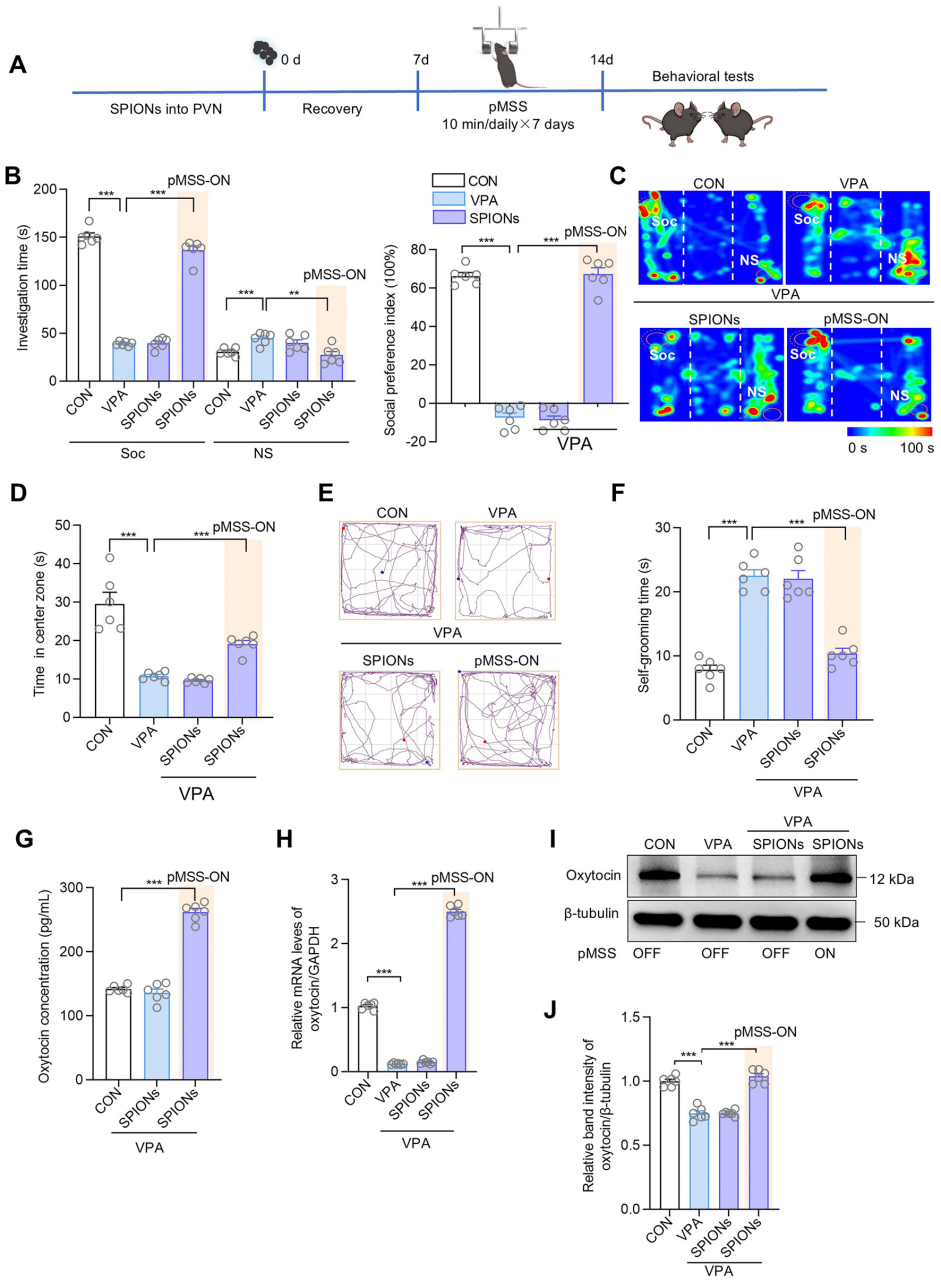
pMSS rapidly ameliorates sociability in mice by promoting endogenous oxytocin release. **A** Schedule of experimental design. **B** Plot of the time investigating social (Soc) *vs* nonsocial (NS) stimuli and social preference index in three-chamber sociability tests of mice, each group *n =* 6 mice. Investigation time: two-tailed Student’s *t* tests; One-way ANOVA: *F* (2, 15) = 373.8 (Soc), *F* (2, 15) = 7.864 (NS). Social preference index: two-tailed Student’s *t* tests; One-way ANOVA: *F* (2, 15) = 268.2. **C** Representative heat maps of mice illustrating the time spent in different locations of the three chambers from social preference tests. Locations of the Soc and NS stimuli are labeled with circles. **D** Open field test, time in the center zone, each group *n =* 6 mice. Two-tailed Student’s *t* tests; One-way ANOVA: *F* (2, 15) = 73.19. **E** Representative trace plot of mice illustrating the time spent in different locations of the open field. **F** Self-grooming test, time of self-grooming, each group *n =* 6 mice. Two-tailed Student’s *t* tests; One-way ANOVA: *F* (2, 15) = 42.31. **G** Results of *in vivo* microdialysis monitoring of oxytocin release at the PVN, each group *n =* 6 mice. One-way ANOVA: *F* (2, 15) = 522.3. **H** The qRT-PCR results of oxytocin in PVN of mice after 7 days of pMSS, each group *n =* 6 mice. Two-tailed Student’s *t* tests; One-way ANOVA: *F* (2, 15) = 4019. **I** Representative immunoblots of oxytocin in the PVN. **J** Western blot for expression levels of oxytocin in PVN of mice after 7 days of pMSS, each group *n =* 6 mice, Two-tailed Student’s *t* tests; One-way ANOVA: *F* (2, 15) = 69.04. All data are represented as the mean ± SD, ^*^P < 0.05, ^**^P < 0.01, ^***^P < 0.001.

We further conducted the behavioral testing after treatment of the ASD model mice by targeting the PVN with pMSS. The SPIONs were bilaterally injected into the PVN of mice using stereotaxic techniques, and the mice were exposed to the pMSS twice daily for 10 min each time (Fig. 6A). The results after one week of treatment are presented in Fig. 6B–F. Notably, continuous one-week treatment with pMSS significantly improved the sociability, reduced the anxiety, and mitigated the repetitive stereotyped behaviors in ASD mice. Then, the brain-derived oxytocin in the mice after magnetic stimulation of the PVN was explored. The microdialysis results demonstrated a local release of oxytocin in the PVN, increasing by up to 262 pg/mL after 10 min of stimulation (Fig. 6G). The RT-qPCR results indicated that the ASD mice showed a low expression of oxytocin, while magnetic stimulation of the PVN reversed this expression, causing a 2-fold increase in oxytocin secretion compared to the wild-type mice (Fig. 6H). The same effect was corroborated by the Western blot analysis (Fig. 6I, J).

**Fig.7:**
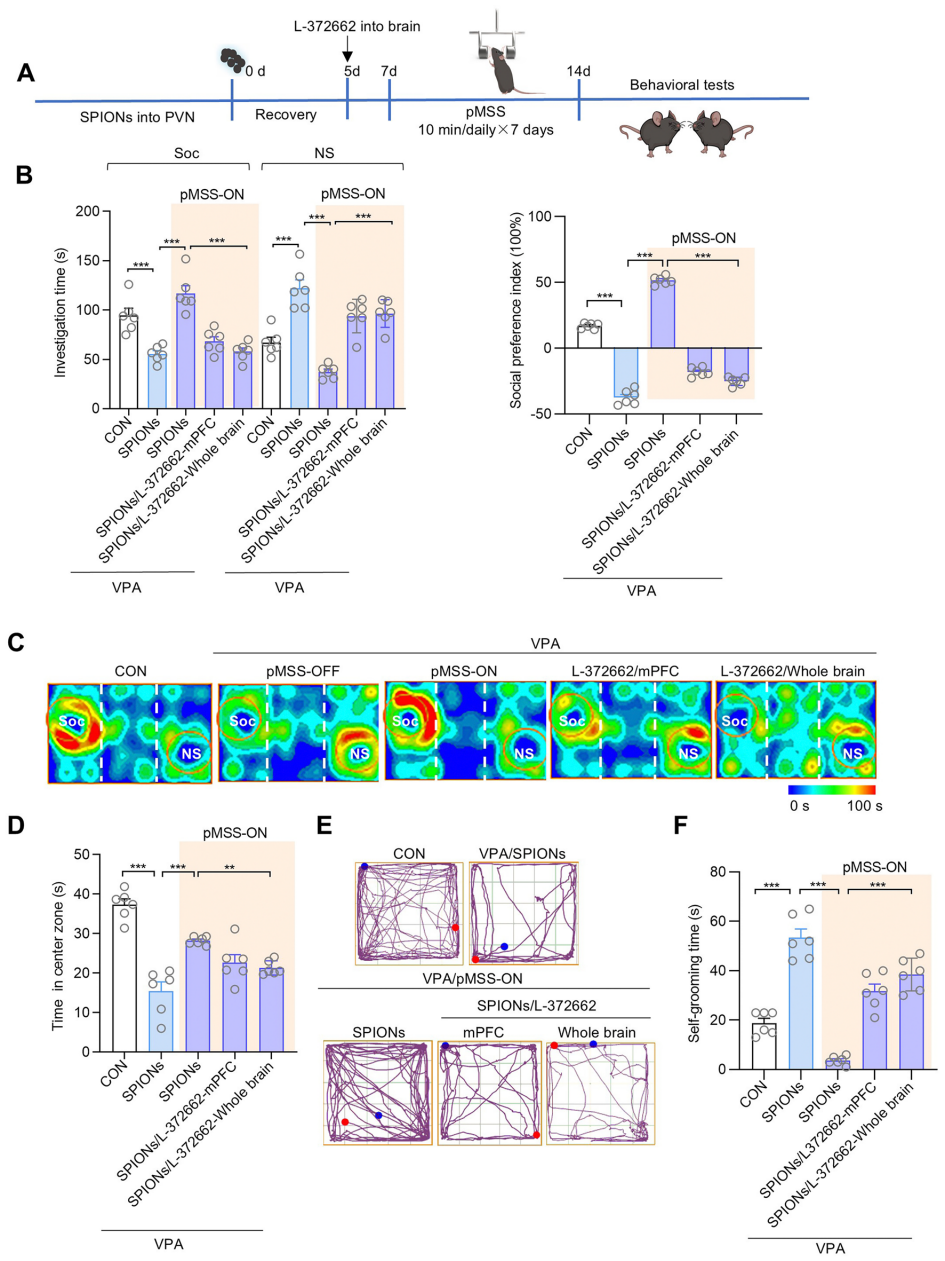
The oxytocin receptor inhibitor L-372662 hinders the process by which 10 Hz-pMSS improves the autism-like behavior in mice. **A** Schedule of experimental design. **B** Plot of the time investigating Soc *vs* NS stimuli and social preference index in three-chamber sociability tests of mice, each group *n =* 6 mice. Investigation time: two-tailed Student’s *t* tests; One-way ANOVA: *F* (2, 15) = 30.27(Soc), *F* (2, 15) = 37.91 (NS). Social preference index: two-tailed Student’s *t* tests; One-way ANOVA: *F* (2, 15) = 1045. **C** Representative heat maps of mice illustrating the time spent in different locations of the three chambers from social preference tests. Locations of the Soc and NS stimuli are labeled with circles. **D** Open field test, time in the center zone, each group *n =* 6 mice. Two-tailed Student’s *t* tests; One-way ANOVA: *F* (2, 15) =8.073. **E** Representative trace plot of mice illustrating the time spent in different locations of the open field. **F** Self-grooming test, time of self-grooming, each group *n =* 6 mice. Two-tailed Student’s *t* tests; One-way ANOVA: *F* (2, 15) = 62.05. All data are represented as the mean ± SD, ^**^P < 0.01, ^***^P < 0.001.

To systematically verify the necessity of the oxytocin system in the improvement of autism-like behavior by 10 Hz-pMSS, this test applied a dual pharmacological intervention strategy: the oxytocin receptor antagonist L-372662 was locally delivered to the mPFC of ASD mice through stereotaxic injection, and the function of oxytocin receptors throughout the brain was inhibited by intracerebroventricular administration. Behavioral tests showed that when the oxytocin signal in the mPFC region was specifically blocked, 10 Hz-pMSS failed to significantly improve the sociability of the mice; while after the group with whole-brain receptor inhibition received stimulation with the same parameters, there was no expected improvement in their social interaction time, anxiety-related behaviors, and repetitive stereotypic behaviors (Fig. 7). These results provide crucial evidence for the therapeutic mechanism by which 10 Hz-pMSS promotes the release of endogenous oxytocin through the activation of the PVN.

**Fig. 8:**
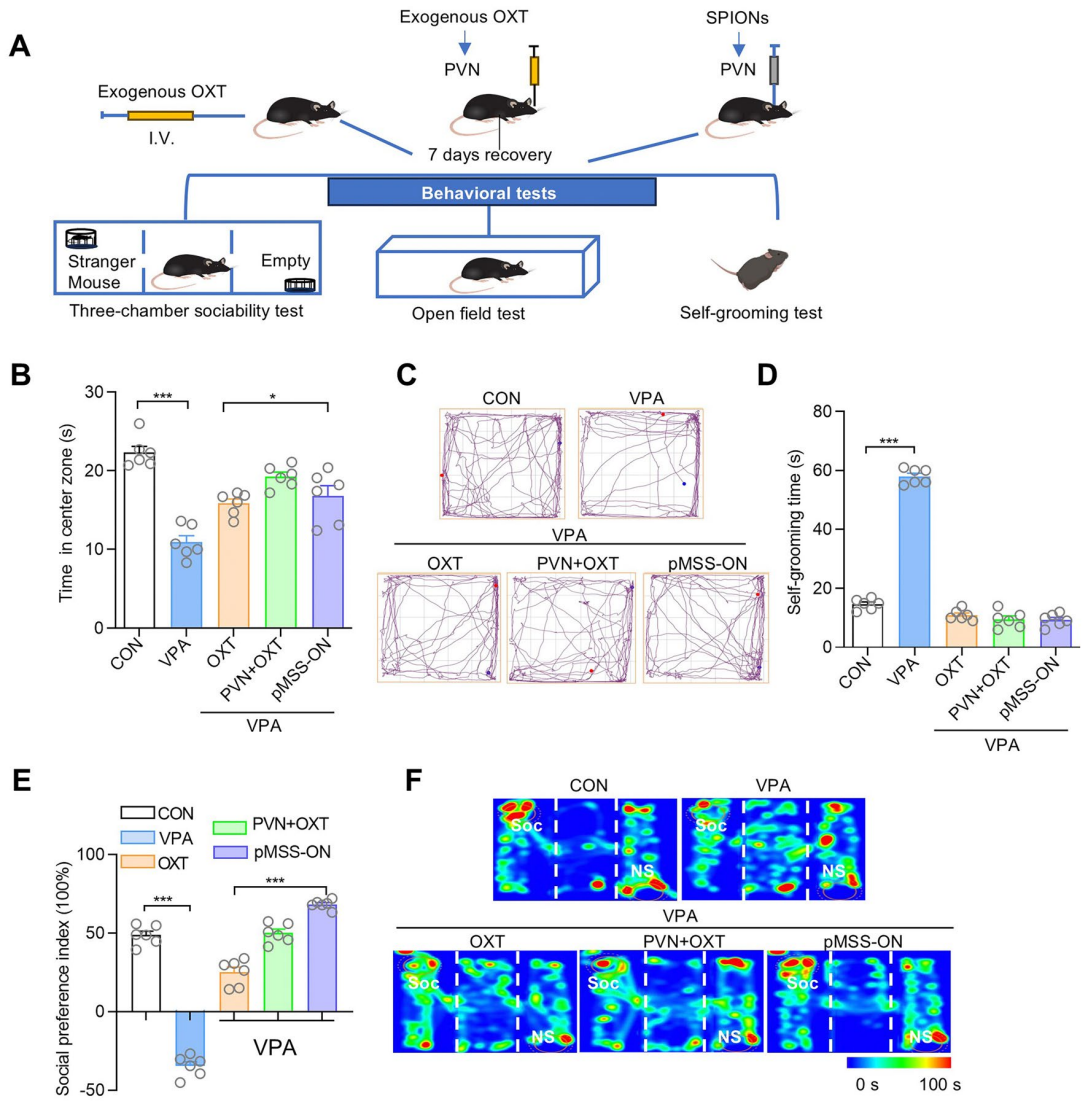
Comparative analysis of exogenous versus endogenous oxytocin on the sociability of mice. **A** Schedule of experimental design. **B** Open field test, time in the center zone, each group *n =* 6 mice. Two-tailed Student’s *t* tests; One-way ANOVA: *F* (2, 15) =3.838. **C** Representative trace plot of mice illustrating the time spent in different locations of the open field. **D** Self-grooming test, time of self-grooming, each group *n =* 6 mice. Two-tailed Student’s *t* tests. **E** Plot of the time investigating Soc *vs* NS stimuli and social preference index in three-chamber sociability tests of mice, each group *n =* 6 mice. Social preference index: two-tailed Student’s *t* tests; One-way ANOVA: *F* (2, 15) = 73.18. **F** Representative heat maps of mice illustrating the time spent in different locations of the three chambers from social preference tests. Locations of the Soc and NS stimuli are labeled with circles. OXT: Oxytocin; VPA+OXT: intravenous injection of OXT; VPA+PVN+OXT: in situ injection of OXT at the PVN; pMSS-ON: pMSS regulates endogenous oxytocin. All data are represented as the mean ± SD, ^*^P < 0.05, ^***^P < 0.001.

Considering that oxytocin had been applied in clinical practice to improve social behaviors, we conducted behavioral testing to compare the influence of endogenous and exogenous oxytocin on mice. The experimental groups included the intravenous administration of oxytocin as used in the clinic, the direct injection of oxytocin into the PVN, and the magnetic stimulation of the PVN (pMSS) *in situ* (Fig. 8A). The behavioral assessments revealed that the anxiety and repetitive stereotypic behaviors were ameliorated in all three groups (Figs. 8B–D). However, it was particularly noteworthy that, compared with the exogenous oxytocin groups, the magnetic stimulation of the PVN group showed a great improvement in the sociability preference index, which was nearly double that of the intravenous administration group (Figs. 8E and F). Here, based on the previous studies showing that the intravenous injection of oxytocin can significantly enhance the sociability of mice at a dosage of 20 mg/kg, we injected ~510 µg and 0.2 µg oxytocin intravenously and directly into the PVN, respectively. By contrast, the amount of oxytocin produced by *in situ* stimulation of the PVN was less than 10.48 pg. These findings implied that SPION-mediated magnetic stimulation of the PVN can be applied as a therapy for social dysfunction disorders, and this is superior to exogenous oxytocin.

## Discussion

This study presents a novel approach to neuromodulation by integrating SPIONs with pMSS, enabling targeted activation of deep brain nuclei such as the PVN. Our findings demonstrate that 10 Hz pMSS effectively enhances PVN neuronal activity, promotes endogenous oxytocin release, enhances neurite outgrowth, and ameliorates autism-like behavior in a VPA-induced mouse model. Below, we contextualize these results within the broader framework of magnetic stimulation technologies, neurobiological mechanisms, and therapeutic potential for ASD.

Traditional rTMS faces limitations in spatial resolution and penetration depth, restricting its ability to target deep brain structures like the PVN [19, 31]. By leveraging SPIONs as mediators, pMSS overcomes these barriers through magneto-confinement effects [32], allowing precise energy delivery to subcortical nuclei. The bidirectional modulation of neuronal activity (inhibition at 1 Hz *vs* excitation at 10 Hz) aligns with established principles of frequency-dependent neuromodulation, yet pMSS achieves this at a significantly lower magnetic field intensity (50 mT *vs* conventional rTMS:1T–3T) [33]. This innovation not only preserves the nature of magnetic stimulation but also enhances its therapeutic specificity, as evidenced by the selective activation of PVN neurons and absence of cortical interference. Moreover, compared to optogenetics and chemogenetics, techniques extensively utilized in basic research, pMSS has its own merits. Optogenetics harnesses light-sensitive proteins (e.g., ChR2, NpHR) along with fiber-optic light stimulation to accurately regulate neuronal activity [34]. Despite its remarkable precision, its translation into clinical applications is impeded by invasiveness and restricted penetration depth. Chemogenetics, which relies on designer receptors uniquely activated by designer drugs (DREADDs) for long-term neuromodulation, is relatively straightforward and less invasive [35]. Nevertheless, it encounters disadvantages such as slow activation and deactivation kinetics and possible off-target effects caused by systemic drug dissemination. However, pMSS uses magnetic nanodrugs to govern neuronal excitability *via* biophysical effects (mechanical force and thermal energy). It provides the benefits of deep-tissue penetration and minimal invasiveness.

The PVN’s role in oxytocin secretion and social behavior regulation is well-documented [22]. Our data reveal that 10 Hz-pMSS induces sustained activation of PVN oxytocinergic neurons, leading to a 2-fold increase in oxytocin secretion and upregulation of downstream signaling pathways (Gαq-PLC-IP3R1-PKC-CD38). These molecular changes are correlated with enhanced dendritic spine density and neurite outgrowth, critical for synaptic plasticity and network integration [36–38]. Notably, the rapid behavioral improvements after one week of pMSS treatment—including increased sociability, reduced anxiety, and diminished repetitive behaviors—mirror the therapeutic effects of exogenous oxytocin administration but with superior efficacy. This suggests that endogenous oxytocin release, localized to PVN projections, may engage distributed oxytocinergic circuits more physiologically than systemic delivery.

While intranasal or intravenous oxytocin administration has shown variable success in ASD clinical trials [39, 40], our findings highlight the limitations of exogenous approaches, such as poor blood-brain barrier permeability and off-target effects. However, pMSS achieves localized oxytocin release within PVN-connected networks, as demonstrated by the failure of oxytocin receptor antagonists (mPFC-specific or global) to rescue behavioral deficits. Moreover, the sustained neuronal activation (≥ 5 h post-stimulation) and biocompatibility of SPIONs (Ferumoxytol^®^) underscore the translational potential of this strategy [41]. Unlike rTMS, which requires high-intensity fields, pMSS operates at mild intensities, minimizing risks of cortical overstimulation or thermal damage.

The bidirectional modulation of neuronal activity (inhibitory at 1 Hz, excitatory at 10 Hz) and structural plasticity (dendritic spine remodeling) described here expands the therapeutic scope of pMSS beyond ASD. For instance, low-frequency protocols could be adapted to suppress hyperactivity in epilepsy or addiction circuits, while high-frequency stimulation might enhance cognitive function in neurodegenerative diseases.

Although our study provides robust preclinical evidence, several limitations deserve attention. First, experiments were conducted in male mice to control for estrous cycle effects. Future work should explore sex-specific responses and hormonal interactions. Second, while acute toxicity assessments of SPIONs yielded reassuring results, long-term safety profiles remain underexplored. Meanwhile, its present spatial resolution, contingent on nanoparticle biodistribution, remains suboptimal. Lastly, translating pMSS technology to clinical settings demands multidisciplinary optimization.

By combining nanotechnology with magnetic stimulation, pMSS represents a paradigm shift in neuromodulation, enabling precise, minimally invasive targeting of deep brain nuclei. Our results not only validate the therapeutic potential of PVN activation in ASD but also establish a framework for developing SPION-mediated therapies for other neuropsychiatric disorders. Future studies should prioritize translational validation, mechanistic exploration of oxytocinergic networks, and integration with closed-loop systems for adaptive stimulation.

## Supplementary Information

Below is the link to the electronic supplementary material.

Supplementary file1 (PDF 1229 KB)

## Data Availability

All data needed to evaluate the conclusions in the paper are present in the paper and/or the Supplementary Material. Additional data related to this paper may be requested from the authors.
