# Supplementary material for: Precise Magnetic Stimulation of the Paraventricular Nucleus Improves Sociability in a Mouse Model of ASD
Source: Neurosci Bull. 2025 Aug 4;41(10):1711–28. doi: 10.1007/s12264-025-01444-x (PMC12495014; doi:10.1007/s12264-025-01444-x)
Supplement: Supplementary file 1 — Supplementary file1 (PDF 1229 KB) [file 12264_2025_1444_MOESM1_ESM.pdf]

Supplemental Information

Video S1: 1 Hz

Video S2: 5 Hz

Video S3: 10 Hz

Video S: Real-time two-photon calcium imaging of PVN neuronal activity under different frequencies of magnetic stimulation. 1 Hz: PVN neuronal activity dynamics in the mouse after 10-min 1-Hz pMSS; 5 Hz: PVN neuronal activity dynamics in the mouse after 10-min 5-Hz pMSS; 10 Hz: PVN neuronal activity dynamics in the mouse after 10-min 10-Hz pMSS.

Note S1

Behavioral tests. Before the experiments, mice were placed in the behavioral test room to acclimate to the environment for at least 2 h. All behavioral tests were applied in the dark circadian period (12:00–18:00).

Body weight: From 7 days after the pups were born, they were weighed every two days, and the weighing continued until they became adults. Finally, the lighter pups were selected.

Eye-opening score: Briefly, compared with wild-type mice, autism-like mice have slower neurodevelopment and can demonstrate delayed eye-opening. The eye-opening score was the following: 0 = both eyes closed, 1 = one eye open, and 2 = both eyes open. Eye-opening was checked once daily from P14 to P18.

Righting reflex test and Hanging wire test: Each mouse was placed on its back and gently held by its four limbs and released. The latency to righting was recorded, such that all four paws were touching the surface. Each mouse underwent the righting reflex test on P9, P11, and P13. The hanging wire test was applied from P14 to P25. Each mouse was placed on a grid wire surface (150 mm × 150 mm, divided into

10-mm grid squares). The surface was inverted, and the latency to fall was recorded. Compared with wild-type mice, autism-like mice have delayed motor function development. Mice with delayed motor development were selected.

Open field test: The male offspring of the tested rats were placed in an open field box and left for 2 min to adapt to the box environment. Then, the total number of crossings of the center zone, the time staying in the center zone, the average speed, and the whole distance were recorded in the following 4 min. Data were analyzed using an ANY-MAZE behavior monitoring system. The autism-like mice spent less time in the center zone and displayed more anxiety.

Self-grooming test: The mice were first put into the open field box to acclimatize for 10 min. Then, the cumulative time spent grooming the face, limbs, body, and tail with their claws or mouth was recorded for 4 min. Autism-like mice groomed themselves for longer periods and had repetitive, stereotyped behaviors.

Three-chamber sociability test: The self-made sociability device consists of a three-chambered box. The mice were left in the device for 10 min to acclimate. The test mouse was placed in the center chamber to adapt for 5 min, and then an unfamiliar mouse marked as strange mouse 1 was placed inside the left cage in the chamber while the right cage in the chamber was empty. The two partitions were quickly opened to make the three chambers interoperable and start testing. The monitoring system kept track of the number, time, distance, and contact time of the experimental mouse with the empty cage and the strange mouse cage for 10 min. Autism-like mice showed less active social time and poor social skills.

Note S2

Ferumoxytol is an ultrasmall superparamagnetic iron oxide (γ-Fe_2O_3) encapsulated with polydextrose-sorbitol carboxymethyl ether (PSC). Generally, the preparation process of the co-precipitation method for the synthesis of ferumoxytol can be divided into three stages: nucleation, growth, and maturation. Firstly, PSC, ferric chloride hexahydrate ($FeCl_3 \cdot 6H_2O$), and ferrous chloride tetrahydrate ($FeCl_2 \cdot 4H_2O$) were dissolved in water, cooled to 10°C, purified with nitrogen, and an excess of ammonium hydroxide (28%)

was added. Subsequently, the mixed solution was heated to 78°C and kept at that temperature for 1 h, and then air was pumped into the mixture. At the end of the reaction, it was purified by ultrafiltration six times using a 100-kDa Molecular Weight Cut-Off membrane, and the final product was obtained by filtration through a 0.2 μm membrane after ultrafiltration. Briefly, ferumoxytol was obtained by the reaction of iron salt with ammonium hydroxide in the presence of PSC.

Ferumoxytol has a molecular weight of ~750 kDa and its molecular formula is $Fe_{5874}O_{8752}C_{11719}H_{18682}O_{9933}Na_{414}$. PSC has a general formula of $C_6H_{11}O_5$-($C_6H_{10}O_5)_{60}$-$C_6H_{13}O_6$, with 14 carboxyl groups ($C_2H_2O_2Na$). Its backbone consists of glucose molecules with intermolecular glycosidic bonds, and some of the carboxy methylated groups and hydrogenated end groups are located at the C4, C3, and C2 positions. The overall size of ferumoxytol is ~17–31 nm, with iron nuclei measuring ~5–10 nm in diameter, and the thickness of the outer coating of PSC is ~1.7 nm. The PSC coating provides a neutral surface for ferumoxytol while stabilizing the internal iron oxides, which significantly reduces the uptake of ferumoxytol by macrophages and the release of free iron prior to uptake by macrophages (an important factor in the promotion of bacterial growth and oxidative stress), thus reducing its potential toxicity and ensuring the safety of rapid administration of relatively high doses.

Table S1 Materials used in the experiment

REAGENT or RESOURCE	SOURCE	IDENTIFIER
**Antibodies**		
Anti-oxytocin (Rabbit monoclonal)	Abcam	Cat# ab212193<br>RRID: AB_2895534
Anti-β-tubulin	Abcam	Cat# ab131205<br>RRID: AB_11156121

Goat Anti-Rabbit IgG H&L (HRP)	Abcam	Cat# ab205718<br>RRID: AB_2819160
Goat anti-Mouse IgG (H+L) Secondary Antibody, HRP	ThermoFisher	Cat # A16066<br>RRID: AB_2534739
Anti-c-Fos antibody [EPR20769]	Abcam	Cat # ab214672<br>RRID: AB_2939046
Anti-Gαq (Mouse monoclonal)	Santa Cruz	Cat# sc-136181<br>RRID: AB_2232474
Anti-IP3R1 (Rabbit Polyclonal)	Invitrogen	Cat# PA1901<br>RRID: AB_2129984
Anti-Phospholipase C beta 1/PLCB1 + Phospholipase C beta 3/PLCB3	Abcam	Cat# 184743<br>RRID: AB_3083510
Anti-PKC alpha (Rabbit monoclonal)	Abcam	Cat# ab32376<br>RRID: AB_777294
Anti-CD38 (Mouse monoclonal)	Proteintech	Cat# 60006-1-Ig<br>RRID: AB_716994
MAP2 antibody [HM-2] - Neuronal Marker	Abcam	Cat# ab11267<br>RRID: AB_297885
Tau antibody [EP2456Y]	Abcam	Cat# ab76128<br>RRID: AB_1524475)
**Bacterial and viral strains**		
rAAV-hSyn-GCaMp6f	Shenzhen Brain Case (China)	BC-0079

AAV1.CamKII. GCaMP6f.WPRE.SV40	Nanjing Brain Observatory, NBO (China)	AV-1-PV3435
rAAV-hSyn-OXT1.0-WPRE-hGH polyA	BrainVTA (Wuhan, China) Co., Ltd	PT-2741
**Chemicals, peptides, and recombinant proteins**		
Oxytocin	MedChemExpress	Cat#HY-17571
Oxytocin receptor antagonist (L372662)	MedChemExpress	Cat#HY-15011
**Critical commercial assays**		
HiScript II OneStep qPCR-PCR-SYBR Green Kit	Vazyme	Cat# Q221-01
OT(Oxytocin) ELISA Kit	Elabscience	Cat#E-EL-0029
FD Rapid Golgi Stain Kit	FD NeuroTechnologies	Cat# PK401
Experimental models: Cell lines		
Neuro-2a	Wuhan Pricella (China)	Cat#CL-0168
**Experimental models: Organisms/strains**		
Mouse: C57BL6/J	Sino-British SIPPR/BK Lab Animal	N/A
**Oligonucleotides**		
Real-time PCR primers	Shanghai GENEray	This paper
**Genes and Species**	**Forward Primer（5’ to 3’）**	**Reverse Primer（5’ to 3’）**
Oxytocin-mouse	GTCTGGTCAAATACTTGCAGGT	CGCGCAGCGAGAAAATGTG
GAPDH-mouse	TTGTCTCCTGTGACTTCAA	CTCTTGCTCTCAGTATCCTT
Microsoft Excel	Microsoft	RRID: SCR_016137

Any-maze	Stoelting	RRID: SCR_014289
GraphPad Prism version 9.0	GraphPad	RRID: SCR_002798
Fiji ImageJ	NIH	RRID: SCR_003070
Photoshop CC	Adobe	RRID: SCR_014199
Imaris	Oxford	RRID: SCR_007370
MatLab	Mathworks	RRID: SCR_001622
**Other**		
Magnetic field generator	Southeast University	This paper
Superparamagnetic iron oxide (SPIO) nanoparticles	Zhengda Tianqing Pharmaceutical Co., Ltd	N/A

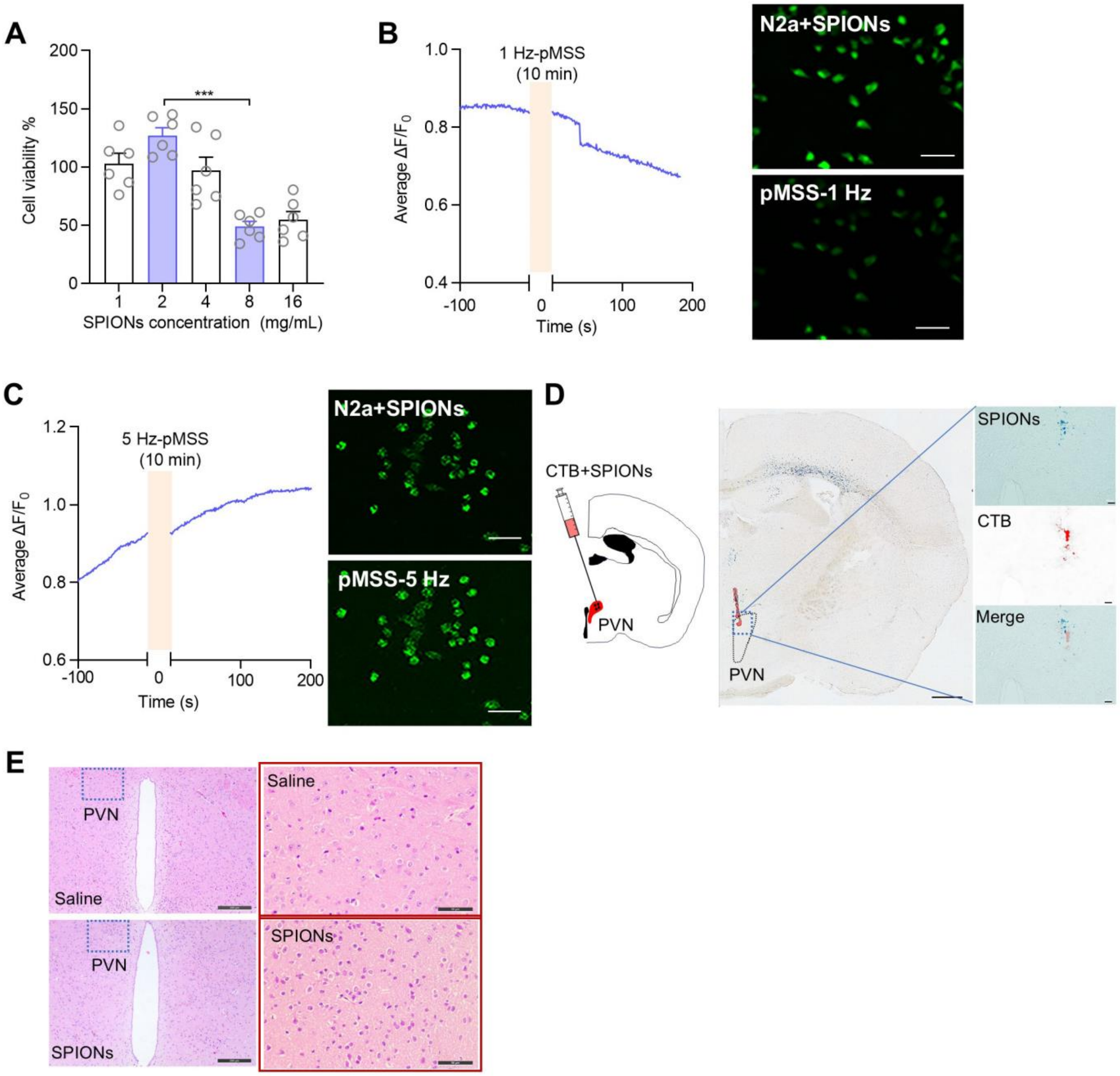

Fig. S1 SPIONs exhibit adequate biocompatibility within the PVN. **A** Cell viability of neurons under different concentrations of SPIONs (one-way ANOVA: F (2, 15) = 23.65, P < 0.001). **B** Average fluorescence intensity change ($\Delta F/F_0$) of N2a cells responding to 1-Hz pMSS. Insets: representative image of N2a cells loaded with Fluo-4 AM before (upper) and after (lower) 1-Hz pMSS (scale bar, 100 μm. n = 25 cells). **C** $\Delta F/F_0$ of N2a cells responding to 5-Hz pMSS. Insets: representative images of N2a cells loaded with Fluo-4 AM before (upper) and after (lower) 5-Hz pMSS (scale bars, 100 μm. n = 24 cells). **D** Cholera

toxin subunit B (CTB) and Perl's staining image of a brain slice from a mouse showing the injection of SPIONs into the PVN (scale bars, 100 μm, 1000 μm). **E** Pathological staining of mouse brain sections after 4 weeks (scale bars, 200 μm, 50 μm).

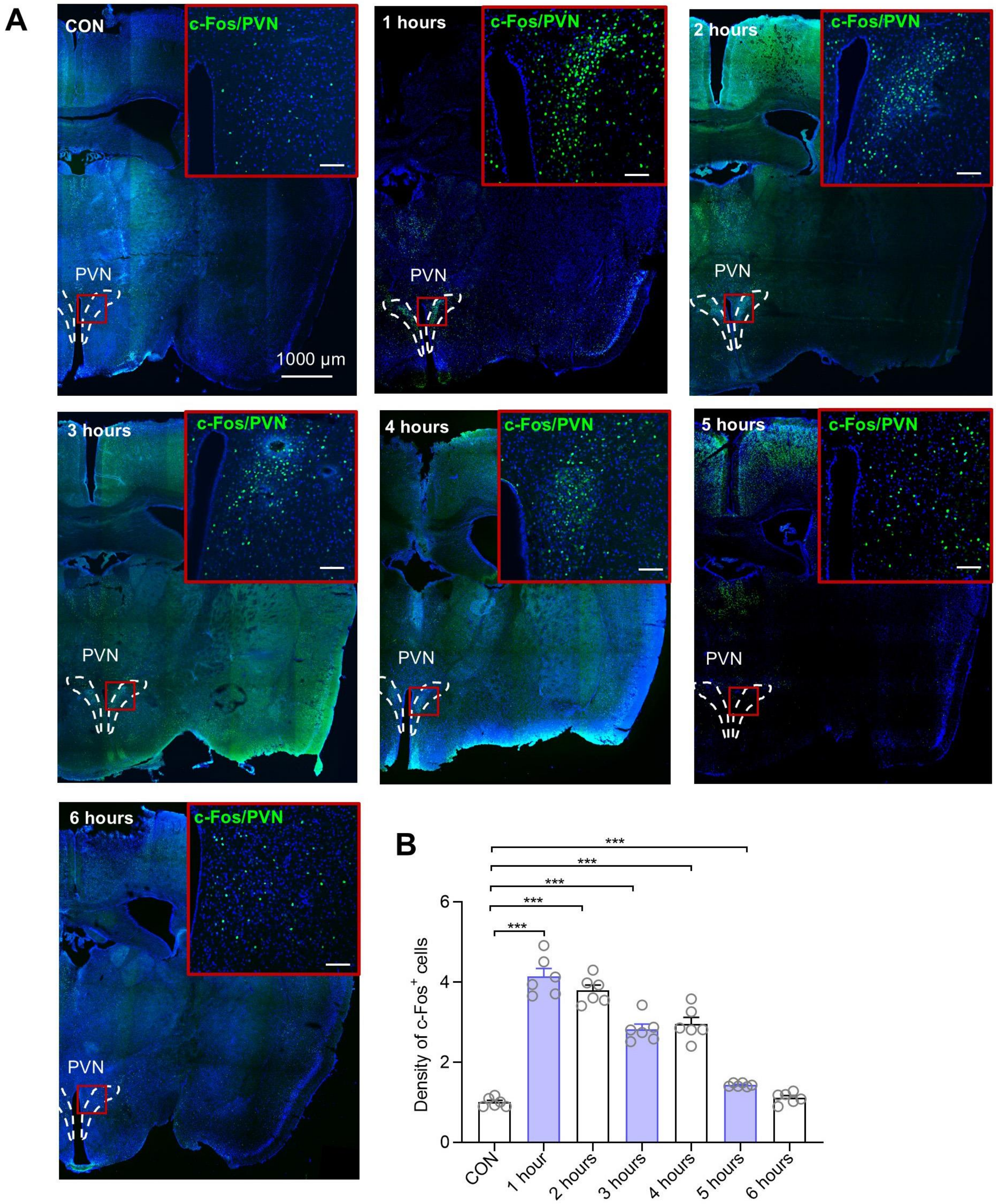

A
CON
c-Fos/PVN
PVN
1000 μm
1 hours
c-Fos/PVN
PVN
2 hours
c-Fos/PVN
PVN
3 hours
c-Fos/PVN
PVN
4 hours
c-Fos/PVN
PVN
5 hours
c-Fos/PVN
PVN
6 hours
c-Fos/PVN
PVN
B
***
***
***
***
***
Density of c-Fos+ cells
0
2
4
6
CON
1 hour
2 hours
3 hours
4 hours
5 hours
6 hours

Fig. S2 10-Hz pMSS efficiently activates neuronal activity in the PVN. **A** Representative images of PVN c-Fos immunofluorescence (coronal sections) after 10 min of pMSS (scale bars, 50 μm, 1000 μm). **B** c-Fos immunopositive cell count in the PVN (n = 6 mice, two-tailed Student's t tests, $^{***}P < 0.001$).

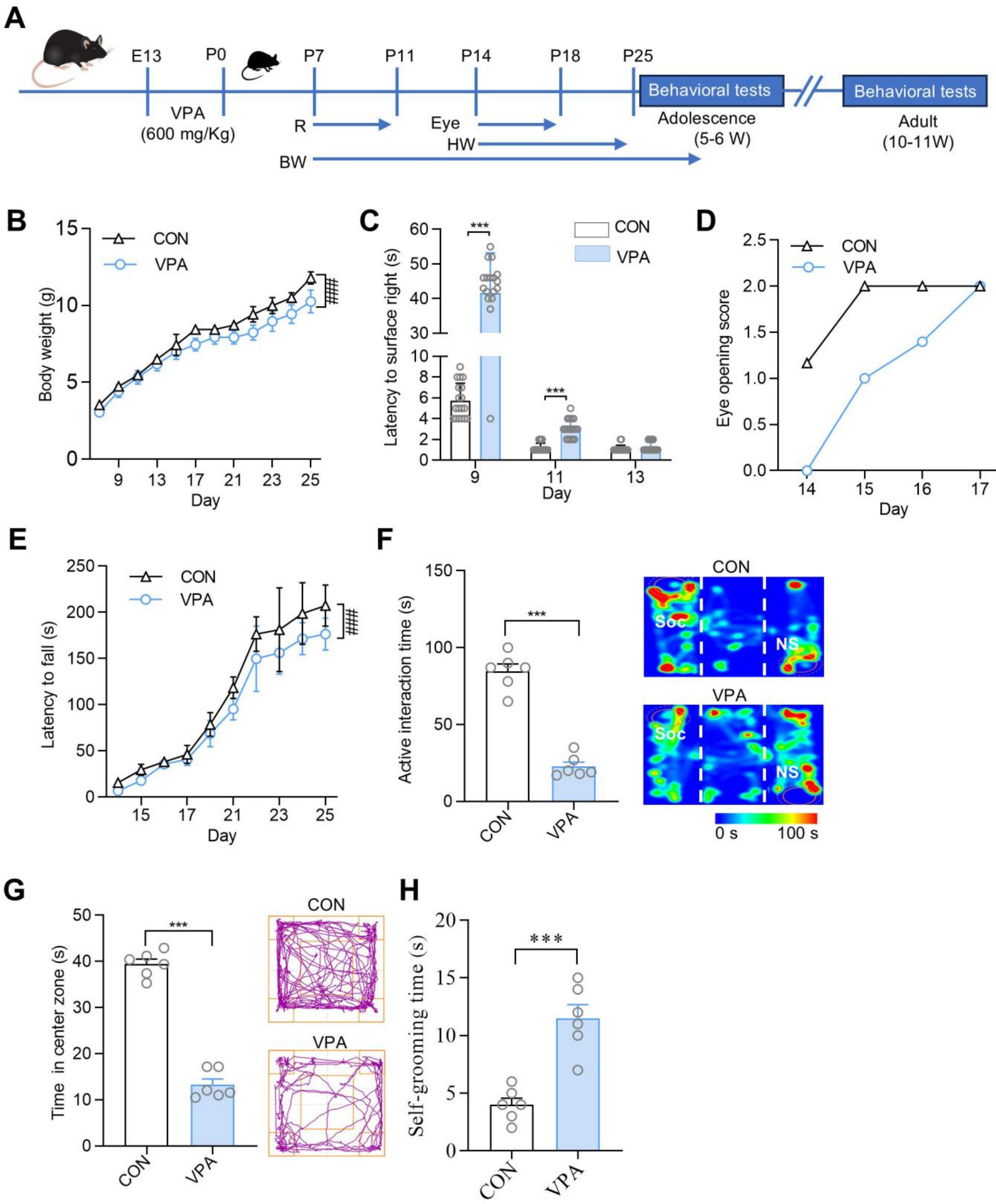

Fig. S3 Constructing a mouse model of ASD. **A** The experimental design. Body maturation and motor function were assessed from P7–P25 (P, Postnatal day). The social interaction test was applied in both adolescence (5–6 weeks) and adulthood (10–11 weeks). The mice were sacrificed to sample whole brains.

BW, body weight test; R, righting Reaction; Eye, eye-opening score; HW, hanging wire test. **B** Body weights (n = 6 mice per group, two-way ANOVA: F (11, 120) = 331.5). **C** Righting reflex time (n = 6 mice per group, two-tailed Student's t test). **D** Eye-opening scores (n = 6 mice per group; 0, both eyes closed; 1, one eye open; 2, both eyes open). **E** Wire hanging times (n = 6 mice per group; two-way ANOVA: F (9, 240) = 364.8). **F** Active interaction times in the three-chamber sociability test (n = 6 mice per group; two-tailed Student's t test). Inset: Representative heat maps illustrating the time spent in different locations of the three chambers from social preference tests. Locations of social (Soc) *vs* nonsocial (NS) stimuli are represented by circles. **G** Open field test, time spent in the center zone (n = 6 mice per group; two-tailed Student's t test). Inset: Representative traces of the open-field test. **H** Self-grooming test, times of self-grooming (n = 6 mice per group; two-tailed Student's t test). All data are represented as the mean ± SD, $^{\#\#\#}P < 0.001$, $^{***}P < 0.001$.

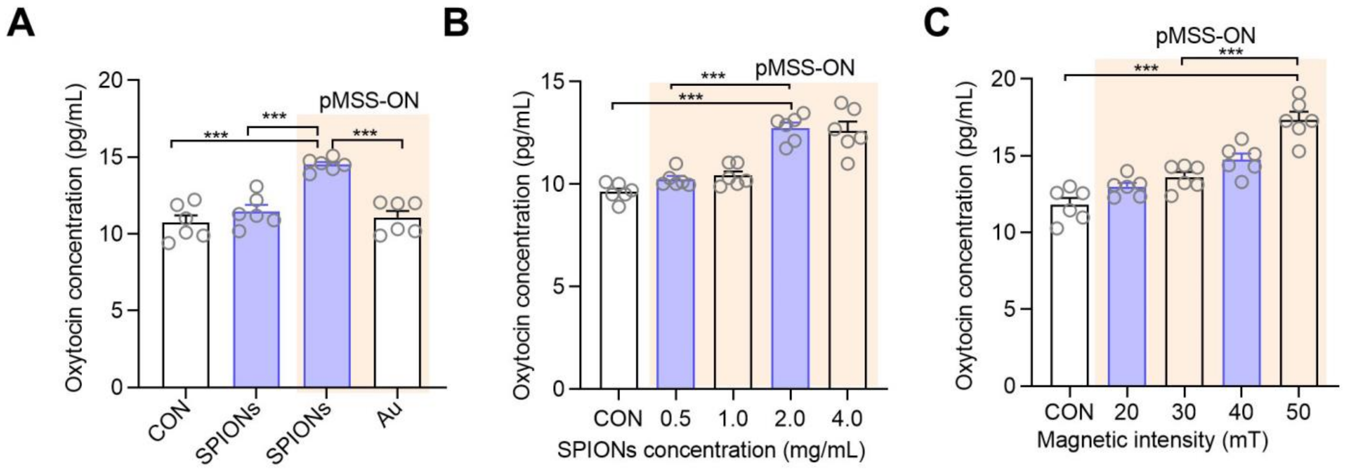

Fig. S4 Appropriate parameters for pMSS to enhance oxytocin release. **A** Effects of different nanoparticles on oxytocin secretion (n = 6 mice per group; two-tailed Student's t test). **B** Effects of different amounts of SPIONs on oxytocin secretion (n = 6 mice per group; two-tailed Student's t tests; one-way ANOVA: F (2, 15) = 41.25). **C** Effects of magnetic field strength on oxytocin secretion (n = 6 mice per group, two-tailed Student's t test; one-way ANOVA: F (2, 15) = 19.95. All data are represented as the mean ± SD, $^{***}P <$ 0.001.
